# Exploring the transmission modalities of Bunyamwera virus

**DOI:** 10.3389/ebm.2024.10114

**Published:** 2024-02-15

**Authors:** Erik A. Turner, Rebecca C. Christofferson

**Affiliations:** Department of Pathobiological Sciences, Louisiana State University, Baton Rouge, LA, United States

**Keywords:** Orthobunyavirus, Bunyamwera, *Aedes aegypti*, environmental transmission, transovarial transmission

## Abstract

Bunyamwera virus (BUNV) (*Bunyamwera orthobunyavirus*) has been found in Sub-Saharan Africa and demonstrated recently as cocirculating with Rift Valley Fever Virus (RVFV). Little is known regarding the breadth of transmission modalities of Bunyamwera. Given its co-occurence with RVFV, we hypothesized the transmission system of BUNV shared similarities to the RVFV system including transmission by *Ae. aegypti* mosquitoes and environmentally mediated transmission through fomites and environmental contamination. We exposed *Ae. aegypti* mosquitoes to BUNV and evaluated their ability to transmit both vertically and horizontally. Further, we investigated the potential for a novel transmission modality via environmental contamination. We found that the LSU colony of *Ae. aegypti* was not competent for the virus for either horizontal or vertical transmission; but, 20% of larva exposed to virus via contaminated aquatic habitat were positive. However, transstadial clearance of the virus was absolute. Finally, under simulated temperature conditions that matched peak transmission in Rwanda, we found that BUNV was stable in both whole blood and serum for up to 28 days at higher total volume in tubes at moderate quantities (10^3–5^ genome copies/mL). In addition, infectiousness of these samples was demonstrated in 80% of the replicates. At lower volume samples (in plates), infectiousness was retained out to 6–8 days with a maximum infectious titer of 10^4^ PFU/mL. Thus, the potential for contamination of the environment and/or transmission via contaminated fomites exists. Our findings have implications for biosafety and infection control, especially in the context of food animal production.

## Impact statement

In many areas where infection control and/or the use of personal protective equipment is not common, there is a risk of transmission via fomites when pathogens are environmentally stable. Our findings demonstrate that BUNV, though an RNA virus, is very environmentally stable and retains infectiousness under certain conditions. Our findings establish the proof-of-principle for this novel transmission modality of environmental contamination of BUNV.

## Introduction

Bunyamwera virus (BUNV) (*Bunyamwera orthobunyavirus*) has been found in Sub-Saharan Africa and demonstrated recently as cocirculating with Rift Valley Fever Virus (RVFV) [1]. RVFV is a mosquito-borne arbovirus of One Health importance. Infected ruminates can experience hemorrhagic fever, spontaneous abortions, and potentially death, especially in young animals [2]. This has not only the obvious animal health implications but can be economically devastating in areas where cattle are a main source of food and/or income [3]. Humans infected with RVFV can also experience hemorrhagic fever, ocular complications, sever flu like symptoms, and death [4]. Originally isolated during an outbreak in the Rift Valley of Kenya in 1930 [5], RVFV has since been found in countries in across Africa and on the Arabian peninsula [6]. As seen with other mosquito-borne viruses, viral transmission of RVFV can often be tied to environmental factors, such as heavy seasonal rainfall and flooding events [7].

During an outbreak of RVFV in Rwanda, BUNV was found to be the etiological agent of abortions in cattle where clinical diagnosis would have attributed such symptoms to RVFV [1]. These two viruses are from the same order (*Bunyavirales*) and are both arboviruses transmitted by mosquitoes [8–13]. BUNV has historically been found in Sub-Saharan Africa. While BUNV can infect humans as well, causing mild symptoms including fever, myalgia/arthralgia, and rashes, the virus has been most often associated with ruminate infection and disease, including spontaneous abortions [1, 14–18]. These symptoms overlap with RVFV, though the transmission patterns of BUNV remain to be fully characterized, and is likely under surveilled. However, given the coincident transmission of RVFV and BUNV, it is reasonable to hypothesize that the BUNV transmission system may share similarities with that of RVFV.

The current understanding of the RVFV transmission cycle includes outbreak/epidemic and maintenance subcycles (Supplementary Figure S1) [9]. The maintenance cycle involves mosquitoes–believed to be including the genus *Aedes–*obtaining infected bloodmeals, subsequently developing a systemic infection, including the ovaries, which results in transovarial transmission [19–21]. *Aedes* spp. eggs are resistant to desiccation [22, 23], allowing for survival through the dry season and, the return of seasonal rains facilitate the hatch of floodwater and container *Aedes* mosquito eggs and the subsequent emergence of infected adults [24]. This mechanism of maintenance through the dry season then seeds a seasonal outbreak, which transitions into the outbreak subcycle, as large numbers of *Culex* spp. mosquitoes hatch after rain events [25, 26]. Additional transmission pathways exist that do not involve the vector. RVFV is a bloodborne pathogen and transmission to humans occurs with handling of infectious fluids from ill livestock, as well as processing raw meat [27–29]. Risk of infection also increased with handling of abortive material [28], which suggests that environmentally mediated infections are an important piece of the transmission puzzle. Adding to the risk of environmentally mediated transmission of RVFV, stability and persistence of infection was noted when originally isolated, as it was found that RVFV is stable in a citrine solution at room temperature for over 7 days [5], and subsequently, this phenomena was replicated at a wide variety of temperatures [30–32], including a potential infection occurring from a sample months old [33]. Further, contamination of larval habitat has been shown to infect larva and emerge as infected adults, indicating that environmentally mediated transmission is a non-negligible part of RVFV transmission risk [34].

*Aedes aegypti*, an important vector of a multitude of human pathogens, is one of the *Aedes* spp. competent for RVFV and BUNV, and several strains were found to be competent vectors of a closely related North American *Orthobunyavirus* species, Cache Valley virus [35–40]. Transovarial transmission of RVFV in *Aedes aegypti* was implicated in the 2007 outbreak in Sudan [41] and has been shown to be successful and stable for three ovarian cycles [20], as well as for a related *Orthobunyavirus,* LaCrosse virus. But transovarial transmission has not been rigorously studied in *Ae. aegypti* for BUNV [42]. Previously, it was demonstrated that BUNV retained infectiousness in cell culture for up to 30 days at 37°C [43]. This sustained persistence led to the consideration of whether BUNV might overlap in the environmentally mediated transmission subcycle of RVFV, as well. Herein, we explore the potential that the BUNV transmission cycle is complex and multi-faceted, like that of RVFV. We investigate the competence of *Aedes aegypti* for vertical and horizontal transmission, and examine factors associated with risk for environmentally mediated transmission under relevant temperature conditions.

## Materials and methods

### Daily Rwandan temperature profile

To simulate relevant temperature conditions, publicly available weather data was mined to generate a daily temperature profile for RVF transmission season in Rwanda, May through July, per the 2018 outbreak [1]. A data scraper collected temperature data in half-hour increments from May 18th through July 12th^,^ 2018, and the temperatures from the same time point each day were averaged to make a single average day. The environmental chamber was then programed to mimic these conditions and a digital thermometer was placed within to confirm the temperature (Supplementary Figure S2). Humidity was maintained at approximately 85% during the experiments roughly matching historical humidity in Rwanda [44].

### Viruses and mosquitoes

BUNV was obtained from the World Arbovirus Reference Center at UTMB had been passaged through suckling mouse cells 47 times and Vero cells 6–8 times prior to the experimentation. Stocks were titered via crystal violet plaque assay and determined to be ∼5.0 × 10^6^ pfu/mL. *Aedes aegypti* mosquito (Rockefeller colony) eggs from were vacuum hatched for 45 min in ddH_2_0 and allowed to emerge as in [45].

### qRT-PCR and plaque assay

Viral RNA extraction was performed via MagMax 96 viral isolation as in [43]. qRT-PCR was performed on a Roche Lightcycler 96, using Quantbio Ultratough master mix and previously published primers and probes [43]. Viral RNA quantification was analyzed via comparison to previously crystal violet plaqued stock from above. Standard crystal violet plaque assays were performed [46]. Virus titer was averaged from countable plaques at each dilution [47].

### qRT-PCR sensitivity assay for mosquito pools

*Ae. aegypti* reared as above were allowed to mature to adults. Adult mosquitoes were cold anesthetized and separated by sex and placed in to 900 µL BA-1 media with 2 stainless steel BBs in a 2 mL locking Eppendorf tube in pools of 10 and homogenized as previously described [45] and a known titer of virus was added to each tube of titers of 10^5^, 10^3^, 10^1^, and 10^0^ pfu/mL. Five replicates per pools sex were performed at each titer.

### Mosquito competence and transstadial transmission

Adult *Aedes aegypti* females, 2 days post emergence (dpe) were offered an infectious blood meal of ratio 2:1 of bovine blood in Alsevers (Hemostat Labs, Dixon, CA, United States) and BUNV infectious supernatant (total infectious titer 1.7 × 10^6^) via the Hemotek feeding apparatus (Discovery Labs, Blackburn, United Kingdom). Engorged females were separated by aspiration and cold anesthetization, then placed in individual 4-ounce containers and provided 10% sucrose solution *ad libitum*. Each container was supplied with egg paper that was moistened daily and collected when eggs were observed for an additional 18 days. Three females died prior to the end of the experiment and were collected and tested for virus in the legs and bodies as described below. At 18 days post infectious bloodmeal, mosquitoes were processed to determine infection status of bodies, legs, and saliva as in [48].

There is mixed evidence for the role of second bloodmeals in enhancing vector competence [48–51]. Thus, a second cohort of mosquitoes was offered an infectious bloodmeal as above at 3–4 days post emergence. A second uninfected bloodmeal was offered starting at 4 days following the first bloodmeal [50] and repeated attempts were made until all females had taken a second bloodmeal (maximum 9 days following first bloodmeal). The mosquitoes were maintained as described above until processed following egg collection at 22–24 post initial, BUNV infectious bloodmeal.

### Environmental mediated transmission of BUNV to *Ae. aegypti* (transstadial transmission)

*Ae. aegypti* were hatched and reared as described above. Fourth instar larva were collected and rinsed in ddH_2_0 and placed into water contaminated with BUNV. Briefly, BUNV supernatant was added to rearing water, and samples were collected at 0–5 days to establish the presence of BUNV RNA [34]. Exposed larva were allowed to mature and were collected as pupa, rinsed, and transferred to a cage for emergence. Pupa were separated based on number of days spent exposed to infected rearing water. Upon emergence, adult mosquitoes were cold anesthetized and separated by sex into pools of up to five based on exposure time. Remaining unemerged individuals were collected at 6 days post contaminated water exposure, rinsed as above, and collated into pools of up to ten for testing by qRT-PCR.

### BUNV environmental stability study

To assess environmental stability of BUNV under environmentally relevant conditions, 110 𝛍L of virus was added to 4.39 mL of two different substrate types–either whole blood or serum for a final concentration of 1 × 105 pfu/mL. The virus-substrate mixture was added to individual units (either falcon tubes or 6-well plates, see Supplementary Methods) and placed into the environmental chamber under Rwanda temperature conditions. Tubes were uncapped and the six-well plate lids lifted to allow air exposure; all units were placed in secondary containment for safety and contamination avoidance. Tubes were sampled at days 0, 1, 7, 21, and 28, while plates were sampled each day until dry. Plates were sampled daily until dry, which occurred between 6 and 8 days. Collected samples were placed into a −80°C freezer until further analysis. Samples were tested starting with the latest timepoint (tubes) or with the day before the plate was dry (Dry Day −1). If a sample was found not to be infectious at a timepoint (I.e., timepoint *X*), the timepoint immediately preceding (i.e., timepoint *X-1*) was tested and this pattern continued until we found the latest time of infectivity. Ten replicates of each unit type were conducted per substrate type. Samples were assayed for stability of RNA via qRT-PCR as above and for infectivity via a growth assay on Vero cells as in [43].

### Statistics

For the sensitivity assay, a Kruskal Wallace non-parametric analysis of variance was used to determine if a difference in detection ability existed between sexes of the pools. A linear regression model was used to evaluate the relationship between the initial input into the mosquito pool and the qRT-PCR output. The limit of detection (LOD) was determined using a qPCR LOD calculation R script (at 95% confidence) [52, 53]. The lowest concentration of consistently detectable RNA was determined by running multiple iterations of a standard curve assay tied to a plaque assay. The plaque assay revealed that maximum stock titer was 5.3 × 10^6^ PFU/mL. The standard curve runs determined that the consistent point at which there was below 95% confidence detection was 5.3 × 10^−1^ PFU/mL. Growth was assessed via non-parametric *t*-test between day 1 and day 7 where a significantly higher titer at day 7 indicated growth and thus infectiousness. To evaluate stability, RNA recovered at each timepoint was compared via a Kruskal-Wallace test where day was a factor; a *post hoc* Dunn’s test (Bonferroni correction) test was performed where appropriate. Additionally, comparisons between RNA recovered from each substrate at the same timepoint or day until dry were done via Wilcoxon Ranked Sum tests. Significance was assessed at the *α* = 0.05 level. Analyses were performed using R Studio (2023.03.1 + 446) and R (4.3.0) and associated packages [54]. Minimum infection rate was determined by calculating the rate of infection as if only one individual per positive pool was infected, as in [55].

## Results

### Sensitivity assay

The qRT-PCR assay used herein was highly sensitive for BUNV in the context of mosquito pool testing (LOD = 6.6 copies/mL with 95% confidence). There was no significant difference in the amount of viral RNA detected in pools of males versus females (Supplementary Figure S3, *p* > 0.05), indicating the assay was sensitive in pools of both sexes. There was only one discrepant result where 5/5 male pools versus 4/5 female pools were positive at the lowest titer of 10^0^ pfu/mL. Combining sexes, at titers of 10^1^ and above, successful detection of virus was 100% (*n* = 10). At 10^0^, BUNV was detected in 90% of pools (*n* = 10, *p* > 0.05).

### *Ae. aegypti* transmission capabilities for BUNV

We found our colony of *Ae. aegypti* were not competent for BUNV and thus not able to horizontally or vertically transmit the virus after a single bloodmeal (*n* = 27). A single mosquito that died at 1 day post exposure tested positive for a midgut infection, but was not included in the analysis as this was more than likely residual RNA from the infectious bloodmeal (midgut titer 1.3 × 10^3^ pfu/mL). Mosquitoes receiving a second bloodmeal also showed no BUNV midgut infection (*n* = 19).

Rearing water contaminated with BUNV remained positive for RNA for the 5 days monitored. Initial quantification of RNA copies were approximately at 10^4.5^ genome equivalents/mL on days 0–1, while this dropped and remained consistent at 10^2.5–3^ for days 2–5. Attempts to quantify BUNV in rearing water via plaque assay were unsuccessful. Despite detection of BUNV RNA, no adult pool tested positive for BUNV. Similarly, a single pool of two pupa that had not emerged by day 6 were also negative for virus (Table 1, see Supplementary Methods). Additionally, larva that had not pupated by day 6 were also pooled and tested. Of the 19 pools, 5 (26%) tested positive for BUNV RNA with low titer (Table 1).

**TABLE 1:** Infection of larva through contaminated water and subsequent transstadial transmission.

Life stage	Number of pools (*n* = 5/pool)	Number of positive pools	Minimum rate of detection (%)	Average quantity recovered (genome equivalents/mL)
Larva	19	5 (26%)	8.33	1.17 × 10^1^
Pupa	1	0	0	NA
Adult	18	0	0	NA

Results from exposure in contaminated rearing water reveal successful uptake of BUNV and/or viral RNA by larva, but unsuccessful transstadial transmission.

### Extracellular stability of BUNV RNA under ecologically relevant conditions

**FIGURE 1:**
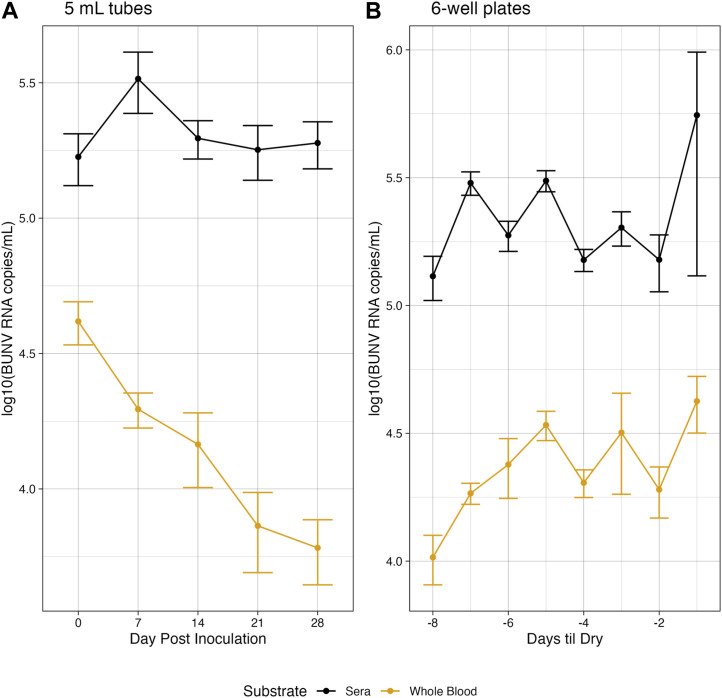
Environmentally stable BUNV. Genomic copies of BUNV recovered at each collection for both higher volume tubes **(A)** and lower volume plates **(B)** over time when exposed to Rwandan environmnetal temperatures.

Under simulated Rwandan conditions, in both whole blood and serum, BUNV RNA was detectable at moderate genome copies/mL up to 28 days in tubes (Figure 1A). The difference in average titer of genome copies between whole blood and sera was statistically significant at all time points (*p* < 0.05). Of interest, both in whole blood and sera, BUNV RNA remained moderately high out to day 28 (6.06 × 10^3^ and 1.89 × 10^5^, respectively). RNA was stable over all days collected for sera. In whole blood a significant decrease in RNA compared to baseline at day 0 was observed starting on day 21, but no further significant decrease was observed between days 21 and 28 (Figure 1A, *p* < 0.05). Unsurprisingly, liquid volume in plates evaporated quickly relative to tubes (Supplementary Figures S4–S5) and plate wells were dry between 6 and 8 days post inoculation. The last day of sampling is referred to as Day Dry—1 (Dry-1, see Supplemental Methods). On average, 4.22 × 10^4^ BUNV genome copies/mL was recovered on Dry-1 for whole blood, while the average recovered genome copies in sera was significantly more (*p* < 0.05) at 1.21 × 10^5^ copies/mL on Dry-1 (Figure 1B). In sera the genome copies were stable as no significant differences were found between the RNA recovered on day 0 and Dry-1 (*p* > 0.05), while the RNA copies recovered in whole blood increased from day 0 to Dry −1 (*p* < 0.05). In a subset of plates (*n* = 5), media was added to dried out wells, collected, and subsequently tested for BUNV RNA (see Supplementary Methods). BUNV RNA was detected in the reconstituted samples, with an average of 6.87 × 10^4^ RNA copies/mL in the reconstituted sera samples, and 6.74 × 10^4^ RNA copies/mL in the reconstituted whole blood samples.

### Infectivity of stable BUNV

**FIGURE 2:**
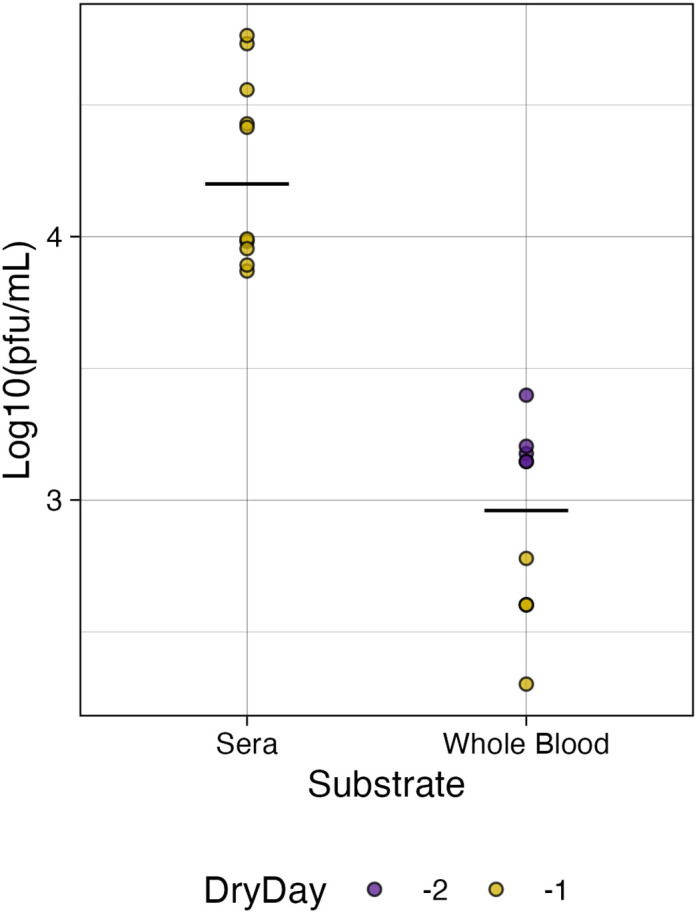
Infectious titer of BUNV in plates. The titer of infectious BUNV recovered from plates on the last day of observed infectiousness was significantly higher for sera than for whole blood (*p* < 0.05) relative to the day the plate was dry (Dry Day).

For whole blood in tubes, the last day of infectiousness was day 28 for 80% (8/10) of samples and day 21 for the remining 20%. In tubes containing sera, the last day of infectiousness was day 21 for 50% (5/10) of replicates while the other 50% retained infectiousness out to day 28. Cytopathic effects (CPE) were observed in all infectious samples. Attempts to plaque sera and whole blood at these later time points were unsuccessful due to the consistency of the samples (see Supplementary Methods, Supplementary Figure S4). In 6-well plates, the range of days where infectiousness was retained was 5–7 days post inoculation but was dependent on the remaining volume in the well (Supplementary Figure S3; Supplementary Table S1). Relative to the first day of no volume, 50% (5/10) of the whole blood replicates retained infectiousness at Dry-1, and 100% at Dry-2. For sera however, all replicates retained infectiousness at Dry −1. We were able to plaque plate samples to further investigate the infectious titer of environmentally stable BUNV on the last day of infectiousness. Overall, whole blood had a significantly lower average infectious titer (1.04 × 10^3^ PFU/mL) compared to sera (2.45 × 10^4^ pfu/mL) (Figure 2, *p* < 0.05). No CPE was observed *in vitro* for any of the reconstituted dried samples, indicating a lack of infectivity despite moderate quantitative recovery of ∼10^4^ RNA copies/mL (see Supplementary Table S2).

## Discussion

BUNV was previously found to co-circulate with RVFV, and as such BUNV has been hypothesized to share aspects of the RVFV transmission cycle [1, 8]. However, the details surrounding the modalities of BUNV transmission remains understudied. RVFV has the potential to be transmitted both vertically and horizontally by *Ae. aegypti* [20, 40]. Further, other Orthobunyaviruses, such Lacrosse virus, Cache Valley virus (a strain of BUNV), and others have been shown to be transmitted by *Ae. aegypti* [35, 36, 42, 56]. We demonstrated that none of the exposed *Ae. aegypti* herein became infected with BUNV via oral exposure, which in turn meant there was no opportunity for ToT. We did show some potential uptake of virus from contaminated larval habitat, but no subsequent transstadial transmission was observed. Thus, there is some potential for refraction to BUNV in *Ae. aegypti* populations as our population did not support infection. As seen with RVFV [34], other *Ae. spp* may be involved in this alternative BUNV transmission modality. Likely there are overlapping ecological factors that contribute to the similarities in the observed transmission of BUNV and RVFV, such as an overall suitability for multiple mosquito species in the region, including seasonal rainfall and other weather patterns that affect mosquito population dynamics in general. However, our data do not rule out the possibility of urbanization of BUNV in other *Ae. aegypti* populations, as another population of this species was found to be moderately competent [39].

These data could also be of use during surveillance efforts during a BUNV disease outbreak. The sensitivity assay demonstrates a high level of sensitivity for this primer set, as 9/10 samples with approximately ∼5 RNA copies per 10 mosquitoes in 1,000 uL and 100% of samples with ∼50 copies tested positive for BUNV via qRT-PCR, with a calculated LOD of 6.6 copies. Previous RT-PCR assays have low-end detections of BUNV RNA at relatively similar levels (700 copies and 2–20 copies per mL) [57, 58]. Further, it was previously found that in *Ae. spp* infected with Cache Valley Virus, an Orthobunyavirus in the Bunyamwera serogroup, the average titer of infectious virus ranged from approximately 3–5 logTCID_50_ [35]. This assay falls well within these limits and is competitive with other available assays, suggesting use as a surveillance tool is plausible.

In areas with RVF transmission, contact with blood and fluids from cattle is highly associated with seropositivity to RVFV [27, 59], indicating the importance of environmentally mediated transmission for the epidemiology of this virus. Our data does support the potential for environmentally mediated transmission of BUNV through blood and/or sera contamination, possibly from processing of meat or contaminated fomites/instruments. In higher volumes (up to 5 mL initial volume in tubes), infectiousness was retained for up to 28 days. At lower volumes over larger surface areas (plates), infectiousness was shorter owing to the shorter time to dried up substrate, but was still retained up to a week. The importance of identifying this potential novel transmission modality of BUNV lies in the potential for education and increased biosafety and infection control practices in at-risk communities, where PPE use is not high [60]. Recently, it was reported that fomite transmission might contribute to the movement of RVFV from rural areas to urban, as cattle may be moved to satiate the demand for meat in human dense areas [61]. This risk is shared by BUNV, as this data demonstrates this similarity in risk of environmentally mediated transmission between RVFV and BUNV. Further, the finding that dried samples were unlikely to harbor infectious virus speaks to the possibility for moisture control as a biosafety measure in areas where this risk exists.

Additionally, the stability of BUNV in whole blood at an environmentally relevant temperature may also inform surveillance efforts. Blood collected from suspected BUNV infected cattle that was unable to be immediately preserved via cooling may still be of use for qualitative surveillance purposes. This also suggests a potential use of blood that was collected indirectly or from meat processing facilities. Butchers and slaughterhouses as a point of surveillance may help inform on the epidemiology of an outbreak due to the concentration of animals to a central location [61]. Furthermore, environmental stability of BUNV in liquid may assist in less conventional surveillance methods for arboviruses, as the potential for wastewater surveillance of other arboviruses has been discussed [62].

While this experiment simulated field conditions in terms of temperature and humidity, additional factors may affect long-term BUNV stability and infectivity that we did not address herein. Both abiotic and biological contaminates found in the environment may alter the stability and infectivity by affecting the length of stability and/or probability of infectiousness over time. Furthermore, the physical environment may also influence BUNV stability, such as the moisture content of soil or rain influencing substrate moisture content which may extend the duration of infectiousness. While this is a limitation of our study, it only further emphasizes a need for a One Health and systems approach when characterizing understudied pathogens.

Given the role of animal and environmental factors and the potential to infect humans, viewing understudied Orthobunyaviruses such as BUNV through a One Health scope is immensely important, as changes animal, human, or the environmental conditions in the area may alter the epidemiology of BUNV. Thus, the data herein is an important addition to the knowledge of this neglected tropical disease and highlights the need for considering atypical transmission routes when cataloging the eco-epidemiology of viruses. It is important that we continue to investigate the interplay between these factors to be prepared to respond to the dynamic pressures of viral transmission and disease outbreaks.

## Data Availability

The original contributions presented in the study are included in the article/Supplementary Material, further inquiries can be directed to the corresponding author.
