# Supplementary material for: Exploring the transmission modalities of Bunyamwera virus
Source: Exp Biol Med (Maywood). 2024 Feb 15;249:10114. doi: 10.3389/ebm.2024.10114 (PMC10954195; doi:10.3389/ebm.2024.10114)
Supplement: Supplementary file 1 [file DataSheet1.pdf]

Exploring transmission modalities of Bunyamwera

EA Turner, RC Christofferson

Supplemental Methods:

Evaporation Study:

Preliminary experiments were conducted to determine the evaporation rate of blood and sera in two different configurations. We observed the evaporation rate of 1), 1.9 ml of whole blood/serum in 2mL centrifuge tubes and 2) 5 mL of whole blood/serum in 6-well plates. Each unit (tube or plate) was placed in an environmental chamber at 28°C (above the maximum temperature of Rwanda) and ~70-80% humidity with box lid and centrifuge tube lids open. Each day the remaining substrate volumes were measured by pipetting up and down several times to homogenize the samples and observing the maximum volume with a serological pipette. This was repeated for a maximum of 28 days or until the unit was dry. Plate wells were dry by day 6, (n=1), day 7 (n=4), or day 8 (n=5) for serum and for whole-blood, by day 7 (n=5) and day 8 (n=5). The last day of sampling is referred to as Day Dry – 1 (Dry-1).

Dry Plate testing:

A subset of plates (n=5) was tested 2-3 days after drying to determine if RNA could still be detected. Briefly, 1 mL of 1x M199 media was added to wells and collected after 20 minutes of intermittent gentle swirling. The media sample was then tested via qRT-PCR for RNA detection and inoculated onto confluent Vero cells to determine infectivity.

Plaque assays attempts with long-sitting whole blood and serum, as well as rearing water:

Crystal violet plaque assays were attempted for both larval rearing water sample and samples retained out to 21 or 28 days in tubes. However, repeated attempts were unsuccessful. In the case of rearing water, the yeast used to feed the larva rendered the water too contaminated for successful plaquing with sample. Attempts at plaquing whole blood at days 21 and 28 days were confounded due to the gelatinous nature of whole blood at these later time points.

Supplemental Figure 1: **Rift Valley Fever virus (RVFV) transmission cycle.** The current understanding of the RVFV transmission cycle suggests two subcycles: the inter-epidemic maintenance cycle driven by Ae. spp. such as Ae. aegypti and the epidemic or outbreak cycle driven by Culex spp. The dotted line pathway represents the potential for environmentally mediated transstadial transmission and its role in the maintenance cycle.

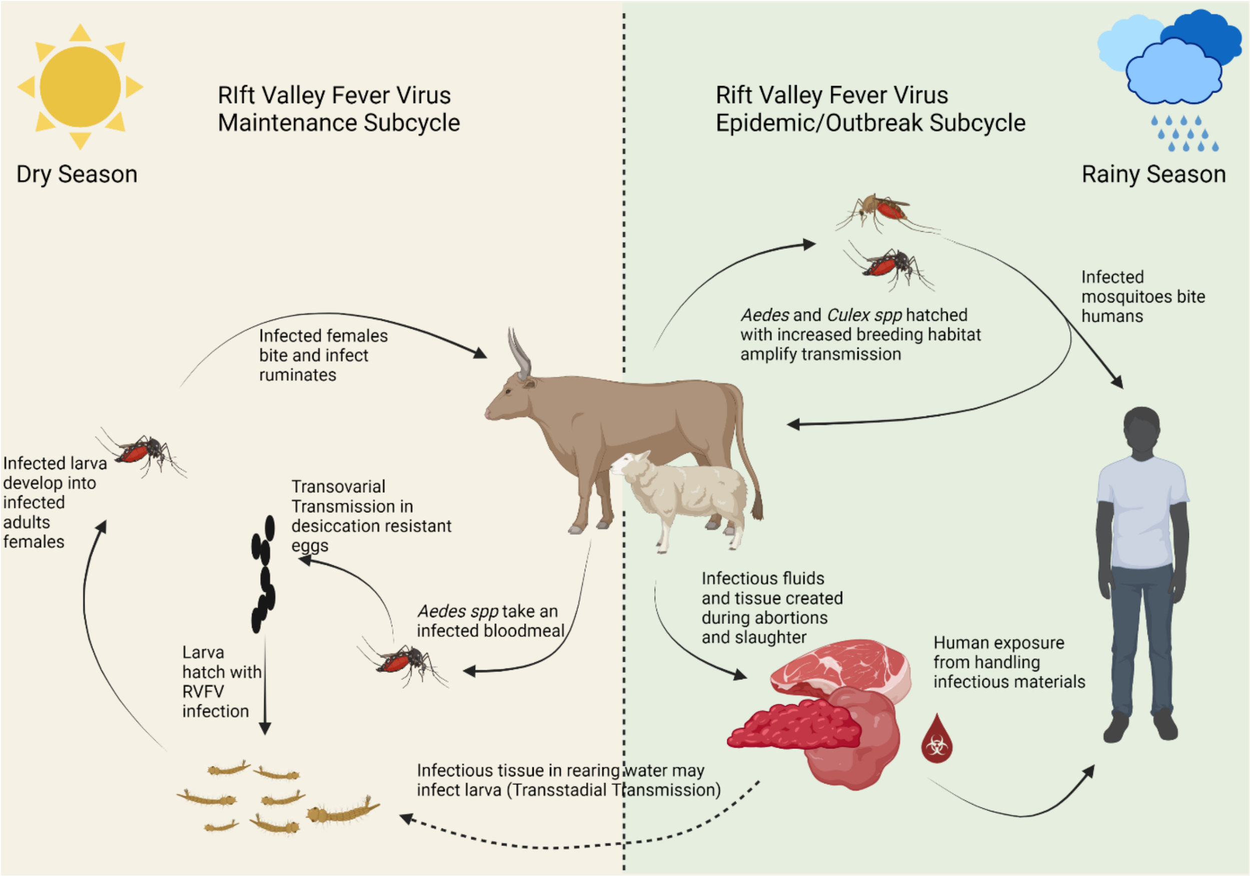

Supplemental Figure S2: Reconstructed Rwandan temperature profile during peak transmission of Rift Valley Fever (May 18th through July 12th, 2018). Rwandan temperature profile constructed from historical temperature data (blue line) and the temperature profile recreated in the environmental chamber (orange line) measured using Kestral Drop2 temperature loggers.

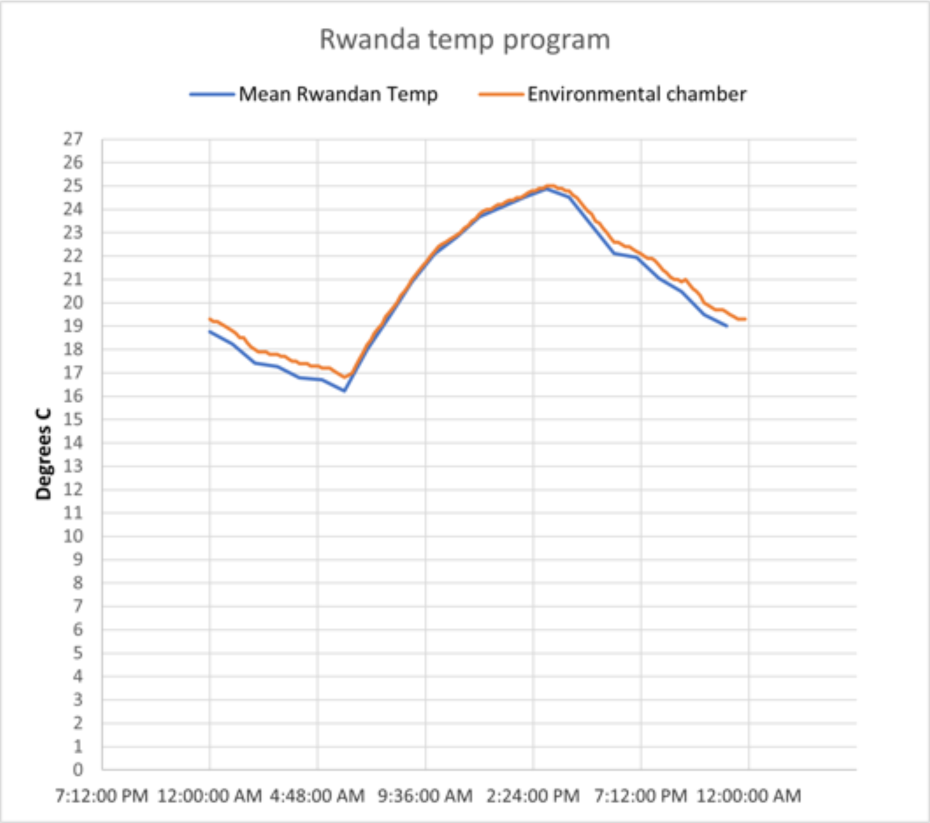

Supplemental Figure S3: Sensitivity of qRT-PCR assay for BUNV at serial dilutions from 10^5 to 10^0 pfu/mL. A predictive fit model using linear regression was established for expected BUNV RNA genome equivalent recovery in mosquito pools (n=10 individuals per pool) (R^2 value of 0.916).

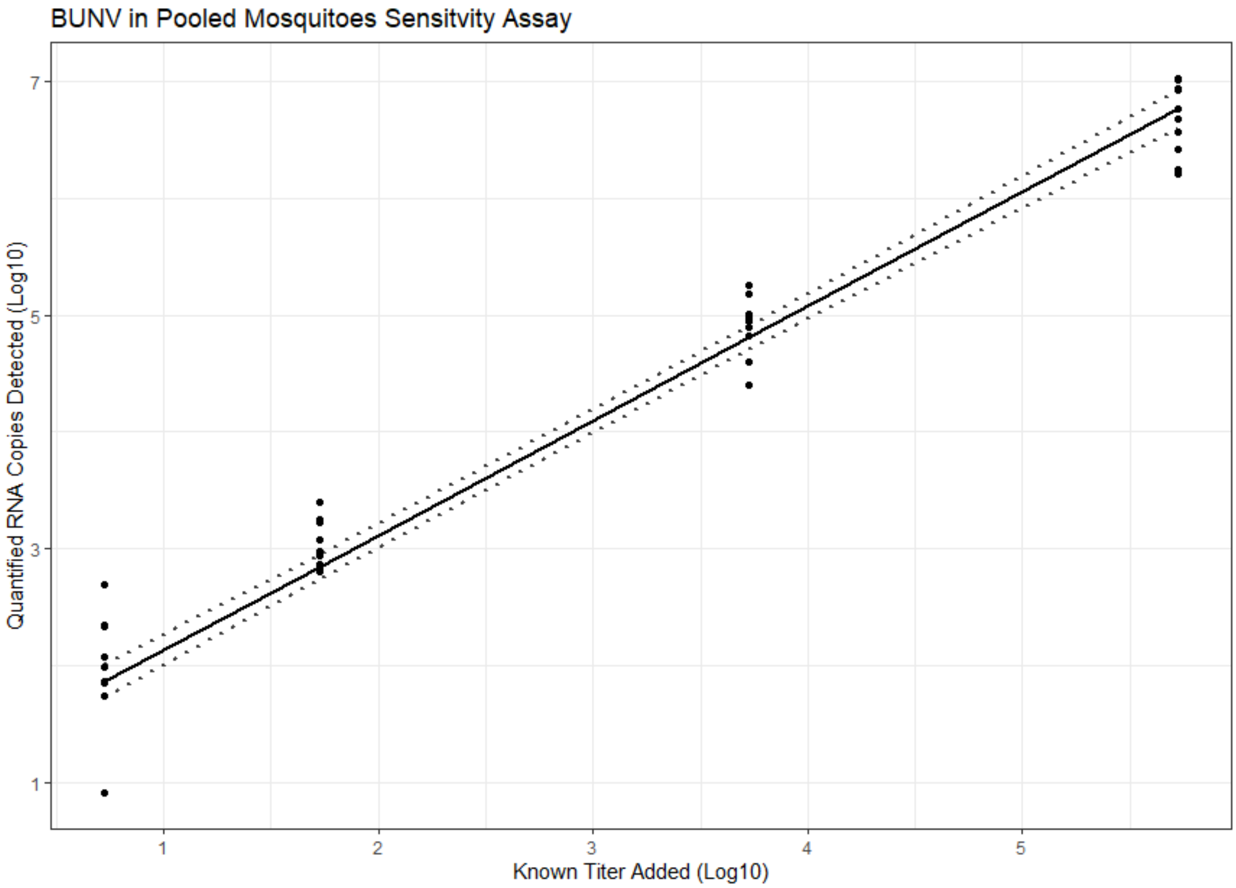

Supplemental Figure S4: Evaporation of serum or whole blood in Eppendorf tubes. Daily volume of serum and whole blood in Eppendorf tubes exposed to the Rwandan temperature profile.

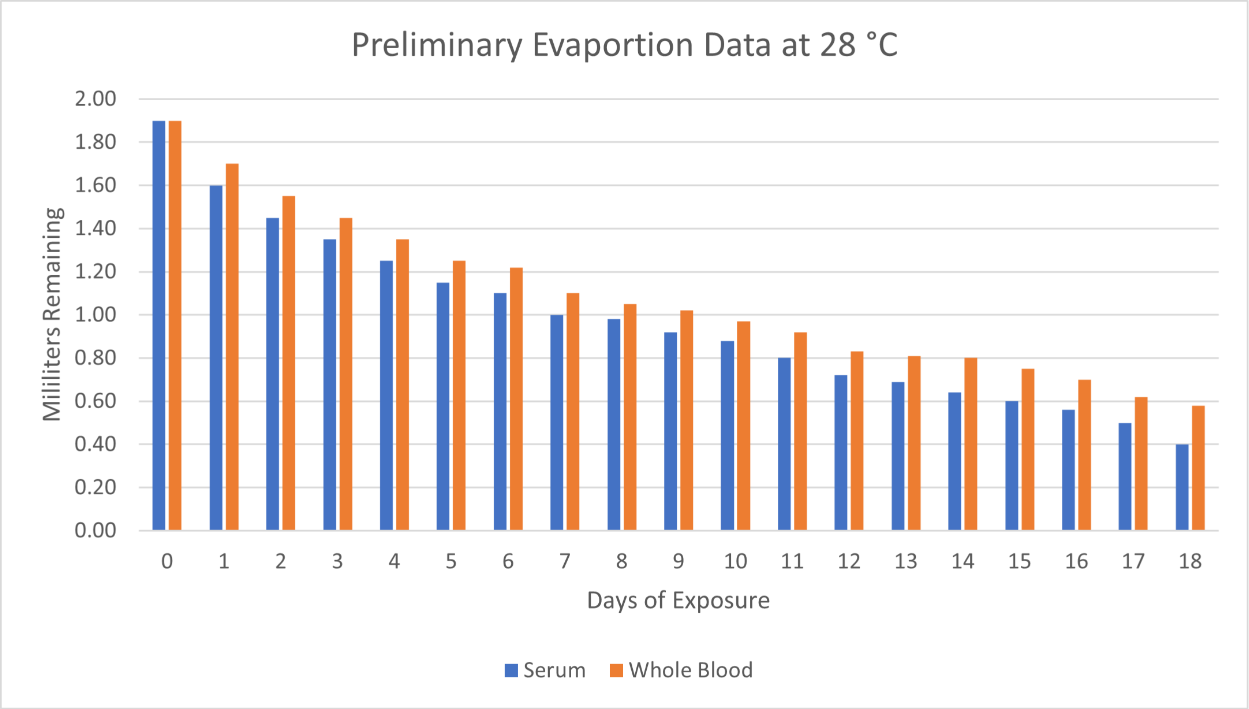

Supplemental Figure S5: Evaporation of serum or whole blood in six well plates. Daily volume (in mL) of serum and whole blood exposed to the Rwandan temperature profile in six well plates.

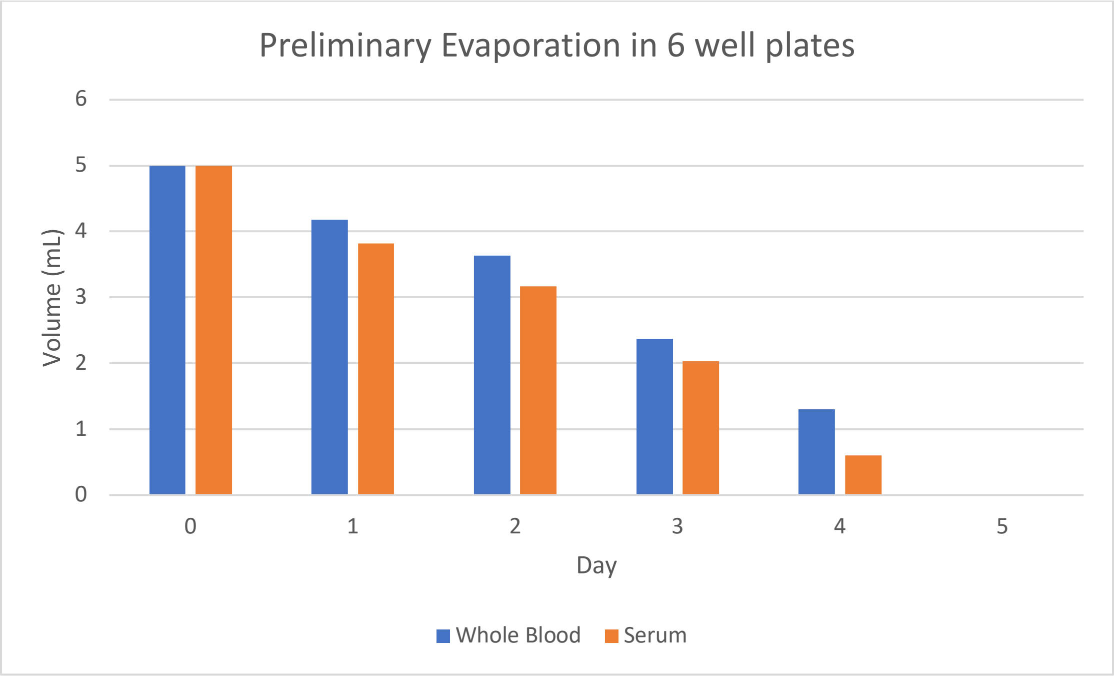

Supplemental Table S1: The last day of infectiousness of samples in 6-well plates relative to the day post inoculation (day of inoculation = day 0). Infectious = 1 means the virus grew in subsequent passage in Vero cells.

Substrate	Day post inoculation	Dry Day	sampleID	Change in Titer 1-7dpi	Infectious
Sera	7	-1	1	7.84E+06	1
Sera	7	-1	2	4.39E+06	1
Sera	7	-1	3	5.46E+06	1
Sera	7	-1	4	5.65E+06	1
Sera	7	-1	5	5.84E+06	1
Sera	6	-1	6	4.69E+06	1
Sera	6	-1	7	5.80E+06	1
Sera	5	-1	8	3.69E+06	1
Sera	6	-1	9	4.32E+06	1
Sera	6	-1	10	5.03E+06	1
Whole Blood	7	-1	1	9.34E+04	1
Whole Blood	7	-1	2	2.44E+05	1
Whole Blood	7	-1	3	1.40E+05	1
Whole Blood	7	-1	4	8.24E+03	1
Whole Blood	7	-1	5	2.46E+05	1
Whole Blood	5	-2	6	7.41E+06	1
Whole Blood	6	-1	6	-3.80E+03	0
Whole Blood	5	-2	7	1.12E+07	1
Whole Blood	6	-1	7	-2.70E+02	0
Whole Blood	5	-2	8	1.23E+07	1
Whole Blood	6	-1	8	-2.55E+03	0
Whole Blood	5	-2	9	1.30E+07	1
Whole Blood	6	-1	9	-7.47E+02	0
Whole Blood	5	-2	10	1.45E+07	1
Whole Blood	6	-1	10	1.80E+03	0

Supplemental Table S2: Observational Growth assay for reconstituted samples. Samples that were reconstituted after drying were tested for the presence of BUNV RNA and found to have an average of 7.68+E04 in whole blood and 7.38E+04 in serum samples. No cytopathic effect (CPE) was observed over a period of 9 days post inoculation in any of the samples despite the presence of moderate quantities of BUNV RNA.

Substrate	Container	Day post inoculation	sample	RNA copies	CPE
Whole blood	Plate	9	6	7.70E+04	None
Whole blood	Plate	9	7	5.99E+04	None
Whole blood	Plate	9	8	5.05E+04	None
Whole blood	Plate	9	9	1.29E+05	None
Whole blood	Plate	9	10	6.77E+04	None
Sera	Plate	9	6	2.62E+04	None
Sera	Plate	9	7	3.76E+04	None
Sera	Plate	9	8	2.35E+05	None
Sera	Plate	9	9	5.88E+04	None
Sera	Plate	9	10	3.43E+04	None
